# Competitive oxidation and ubiquitylation on the evolutionarily conserved cysteine confer tissue-specific stabilization of Insig-2

**DOI:** 10.1038/s41467-019-14231-w

**Published:** 2020-01-17

**Authors:** Zhang-Sen Zhou, Mei-Xin Li, Jie Liu, Hengwu Jiao, Jing-Ming Xia, Xiong-Jie Shi, Huabin Zhao, Liping Chu, Jingrong Liu, Wei Qi, Jie Luo, Bao-Liang Song

**Affiliations:** 1 Hubei Key Laboratory of Cell Homeostasis, College of Life Sciences, Wuhan University, Wuhan, 430072 China; 2 School of Life Science and Technology, ShanghaiTech Universiy, Shanghai, 201203 China

**Keywords:** Biochemistry, Evolution

## Abstract

Insig-2 is an ER membrane protein negatively controlling lipid biosynthesis. Here, we find that Insig-2 is increased in the tissues, including liver, but unaltered in the muscle of *gp78*-deficient mice. In hepatocytes and undifferentiated C2C12 myoblasts, Insig-2 is ubiquitylated on Cys215 by gp78 and degraded. However, the C215 residue is oxidized by elevated reactive oxygen species (ROS) during C2C12 myoblasts differentiating into myotubes, preventing Insig-2 from ubiquitylation and degradation. The stabilized Insig-2 downregulates lipogenesis through inhibiting the SREBP pathway, helping to channel the carbon flux to ATP generation and protecting myotubes from lipid over-accumulation. Evolutionary analysis shows that the YECK (in which C represents Cys215 in human Insig-2) tetrapeptide sequence in Insig-2 is highly conserved in amniotes but not in aquatic amphibians and fishes, suggesting it may have been shaped by differential selection. Together, this study suggests that competitive oxidation-ubiquitylation on Cys215 of Insig-2 senses ROS and prevents muscle cells from lipid accumulation.

## Abstract (web summary)

The protein Insig-2 negatively regulates lipid biosynthesis and is short-lived in liver cells but stable in muscle cells. Here, the authors show that in muscle cells producing reactive oxygen species, there is increased oxidation compared to ubiquitination of Insig-2, stabilising Insig-2 and reducing lipid biosynthesis.

## Introduction

Cholesterol, the most abundant sterol, is essential for membrane function in mammalian cells and its metabolism is tightly regulated. Cellular cholesterol can be synthesized from acetyl-CoA through the mevalonate pathway and obtained through receptor-mediated endocytosis of lipoproteins. Insulin-induced gene (Insig)-1 and Insig-2, two closely related ER membrane proteins, are key negative regulators controlling lipid biosynthesis and uptake through acting on the sterol-regulated maturation of sterol regulatory element-binding proteins (SREBPs) and degradation of HMG-CoA reductase (HMGCR)^1–3^. When cellular sterol level is high, Insigs bind to the SREBP/SREBP cleavage-activating protein (SCAP) complex, blocking its transport from the ER to Golgi and subsequent proteolytic cleavage of the SREBP precursors^4^. As a result, the transcription of lipid biosynthetic genes is downregulated. High concentrations of sterols also promote Insigs and associated ubiquitin ligase gp78 to bind HMGCR, leading to ubiquitylation and proteasomal degradation of HMGCR^5–8^. Genetic deletion of *Insigs* resulted in robust increases in mRNA abundance of lipogenic genes and HMGCR protein level, as well as overaccumulation of cholesterol and triglyceride in the liver.

Insig-1 is a short-lived protein in cultured cells. A high concentration of cholesterol promotes SCAP to bind and stabilize Insig-1, whereas cholesterol depletion dissociates the SCAP-Insig-1 complex and accelerates Insig-1 ubiquitylation on the K156 and K158 residues by gp78 and degradation^9–11^. Insig-2, albeit sharing about 59% of sequence identity with Insig-1, is stable in some cultured cells including Chinese hamster ovary (CHO) and human fibroblast SV589 cells^12,13^. However, our previous work showed that Insig-2 was stabilized and accumulated in *gp78*-deficient mouse liver^14^. The underlying mechanism of tissue-specific Insig-2 stabilization is unknown.

In this study, we generate whole-body *gp78* knockout mice and find that the protein level of Insig-2 is markedly increased in liver, adipose tissue and kidney. However, Insig-2 is surprisingly unaltered in muscle. Using hepatocytes, we demonstrate that Insig-2 is ubiquitylated on Cys215 by gp78. On the other hand, oxidization of Cys215 in myotubes by reactive oxygen species (ROS) outcompetes ubiquitylation and protectes Insig-2 from degradation, which prevents muscle cells from lipid overaccumulation. Intriguingly, the YEC_215_K tetrapeptide of Insig-2 is evolutionarily conserved in amniotes but not in amphibians or fishes, which bear less oxygen and ROS. Together, our study reveals a tissue-specific regulation of Insig-2 stability through oxidation and ubiquitylation on Cys215 residue and implicates a link between metabolic oxidative state and lipid biosynthesis.

## Results

### Insig-2 is unchanged in the muscle of *gp78*-deficient mice

**Fig. 1:**
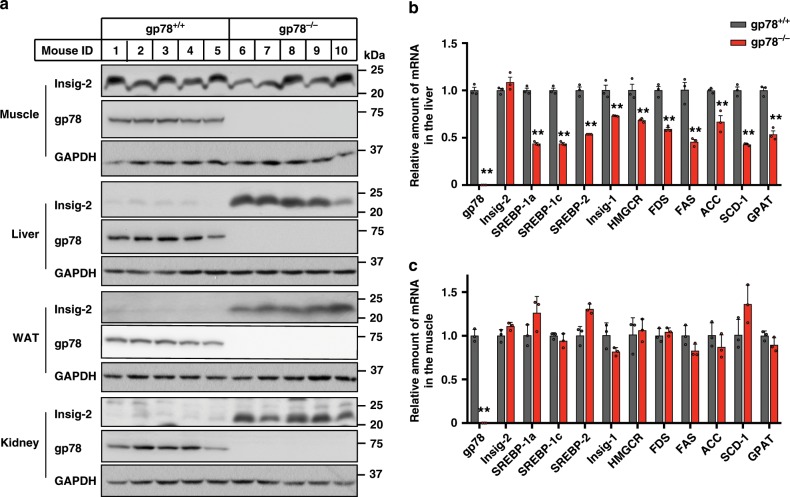
Ablation of *gp78* increases Insig-2 protein level in the liver, WAT and kidney but not in the muscle. Male *gp78*^*−/−*^ mice and their wild-type (WT) littermates (*gp78*^*+/+*^) (8-week-old, *n* = 5 per group) were sacrificed and harvested for immunoblotting (**a**) and RT-qPCR (**b**, **c**) analysis. **a** Expression of indicated proteins in different tissues. **b**, **c** The mRNA levels of indicated genes in the liver (**b**) and muscle (**c**). Data are presented as mean ± s.d. **p < 0.01,unpaired two-tailed Student’s *t* test.

We first generated whole-body *gp78* knockout mice (*gp78*^*−/*−^) by crossing mice with loxP sites flanking the exon 5 and 8 of *gp78* (*gp78*^*f/f*^) with those expressing Cre recombinase under the control of a cytomegalovirus (CMV) promoter. The *gp78*^*−/*−^ mice were viable and appeared normal. We then compared the Insig-2 protein levels in various tissues between *gp78*^−*/*−^ mice and their wild-type (WT) littermates. Ablation of *gp78* increased Insig-2 protein in the liver, white adipose tissue and kidney (Fig. 1a). Surprisingly, Insig-2 protein amount remained unaltered in the skeletal muscle of *gp78*^−*/−*^ mice compared with that of WT controls (Fig. 1a). But the Insig-2 protein was elevated in the heart of *gp78*-deficient mice (Supplementary Fig. 1a). Since Insig-2 negatively regulates SREBP processing^1^, we next analyzed the expression of an array of SREBP target genes in the liver and skeletal muscle by quantitative polymerase chain reaction (qPCR). Except for *Insig-2*, the mRNA abundance of *Srebp-1a*, *Srebp-1c*, *Srebp-2*, *Insig-1*, as well as the genes involved in cholesterol synthesis (*Hmgcr* and farnesyl diphosphate synthase *[Fds]*) and fatty acid and triglyceride (TG) synthesis (fatty acid synthase *[Fas]*, acetyl-CoA carboxylase *[Acc]*, stearoyl-CoA desaturase-1 *[Scd-1]*, glycerol-3-phosphate acyltransferase *[Gpat]*) were all significantly downregulated in the liver of *gp78*^−*/*−^ mice (Fig. 1b). By contrast, the mRNA levels of all examined genes were similar in the skeletal muscle of *gp78*^−*/−*^ mice and WT controls (Fig. 1c).

### Cys215 of Insig-2 is ubiquitynated by gp78 in hepatic cells

**Fig. 2:**
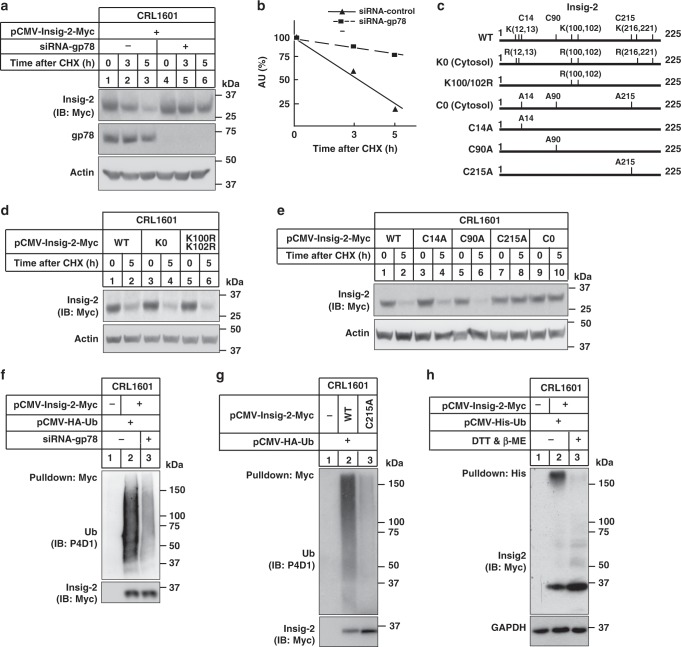
Ubiquitylation and degradation of Insig-2 requires gp78 and the Cys215 residue. **a** CRL1601 cells were transfected with the plasmid expressing Myc-tagged Insig-2 and indicated siRNAs. Cells were then treated with 100 μM cycloheximide (CHX) for indicated periods and harvested for immunoblotting analysis. **b** Densitometric analysis (*n* = 3 independent quantification) of Insig-2 levels in (**a**). AU arbitrary unit. **c** Schematic of human Insig-2 protein and various mutants. **d**, **e** CRL1601 cells were transfected with plasmids expressing different Insig-2 variants in (**c**), and then treated without or with 100 μM CHX for 5 h followed by immunoblotting analysis. **f** CRL1601 cells were transfected with indicated plasmids and siRNAs, and treated with 10 μM MG132 for 5 h. Cells were harvested for ubiquitylation assay. **g** CRL1601 cells were transfected with indicated plasmids and treated with 10 μM MG132 for 5 h. Cells were harvested and lysed in denaturing IP buffer and subjected to ubiquitylation assay. **h** CRL1601 cells were transfected with indicated plasmids and treated with 10 μM MG132 for 5 h. Cells were harvested, lysed in denaturing IP buffer followed by incubation with the Ni-NTA beads. Precipitates were treated with (+) or without (−) DTT (75 mM) and β-ME (1.5%) prior to immunoblotting analysis.

It is known that gp78 promotes ubiquitylation and degradation of Insig-1 (ref. ^10^). To investigate whether gp78 is required for Insig-2 degradation, we expressed Myc-tagged Insig-2 in control and *gp78*-knockdown CRL1601 cells, and performed a cycloheximide (CHX) chase assay. A rapid degradation of Insig-2 was observed in control RNAi cells, with a half-life of ~3.5 h (Fig. 2a, b). However, about 80% of Insig-2 protein remained stable even after 5 h of CHX treatment upon *gp78* deficiency (Fig. 2a, b).

Human Insig-2 is 59% identical to Insig-1 and proteasomal degradation of Insig-1 requires ubiquitylation of Lys156 and Lys158 (equivalent to Lys100 and Lys102 of human Insig-2)^9,15,16^. To investigate whether the Lys100 and Lys102 residues are responsible for gp78-mediated degradation of Insig-2, we mutated both lysines to arginines (K100R/K102R) and found the mutants degraded as fast as the WT one (Fig. 2c, d). In fact, substituting all cytoplasmic lysines for arginines (K0) did not affect Insig-2 degradation as revealed by CHX chase analysis (Fig. 2c, d). Because ubiquitin can also be covalently attached to the SH moiety of cysteine residues^17,18^, we next replaced the cysteine residues on the cytoplasmic side with alanines (Fig. 2c). Insig-2 carrying all three cysteine mutations (C0) remained steady at 5 h of CHX treatment (Fig. 2e). In particular, the C215A mutation but not other single cysteine mutations (C14A and C90A) effectively prevented Insig-2 degradation (Fig. 2e). Together, these results suggest that Cys215 is required for gp78-mediated degradation of Insig-2 in CRL1601 cells.

To exclude the possibility that the C215A mutation impairs the interaction between Insig-2 and gp78, we immunoprecipitated transfected WT Insig-2 and Insig-2 (C215A) proteins using the anti-Myc coupled agarose. As shown in Supplementary Fig. 1b, comparable amounts of gp78 were detected co-immunoprecipitated with WT and mutant Insig-2 proteins. To assess whether the C215A mutation affects Insig-2 function, we examined Insig-mediated degradation of the overexpressed HMGCR protein. Both WT and C215A mutant supported sterol-regulated degradation of HMGCR (Supplementary Fig. 1c). Together, these results suggest that the C215A mutation neither alters Insig-2 binding to gp78 nor impairs Insig-2 function in mediating HMGCR degradation.

We next examined whether Insig-2 could be ubiquitylated by gp78. The Insig-2 protein was immunoprecipitated from control or *gp78*-knockdown cells in the presence of proteasome inhibitor MG132 followed by immunoblotting analysis with the anti-ubiquitin antibody. Compared with the high-molecular-weight smear in control cells, substantially reduced ubiquitylation was detected upon *gp78* knockdown (Fig. 2f). It has been known that UBE2G2 is the E2 cooperating with gp78^19^. Knockdown of UBE2G2 dramatically decreased the Insig-2 ubiquitylation (Supplementary Fig. 1d, e). To exclude the possibility that ubiquitylation may take place on Insig-2-associated proteins, we pulled down WT and C215A forms of Insig-2 in the denaturing conditions that disrupt all protein interactions. The ubiquitylation of WT but not mutant Insig-2 was detected (Fig. 2g). Next, we compared the ubiquitylation level of Insig-2 in the absence or presence of dl-Dithiothreitol (DTT) and beta-mercaptoethanol (BME). These reducing agents can disrupt the thioester bond between ubiquitin and cysteine residues, producing un-ubiquitylated proteins with smaller molecular mass^18^. Indeed, addition of DTT and BME apparently downshifted the transfected Insig-2 protein to its original molecular weight of ~33 kDa (Fig. 2h). In addition, we treated the Insig-2 IP samples with DTT/BME and hydroxylamine. DTT/BME and hydroxylamine could down-shift the ubiquitin signal, suggesting that they broke the thioester bond between cysteine and ubiquitin molecules (Supplementary Fig. 1f). Together, these results demonstrate that Insig-2 is ubiquitylated on Cys215 by gp78.

### gp78 promotes Insig-2 degradation in undifferentiated myoblasts

**Fig. 3:**
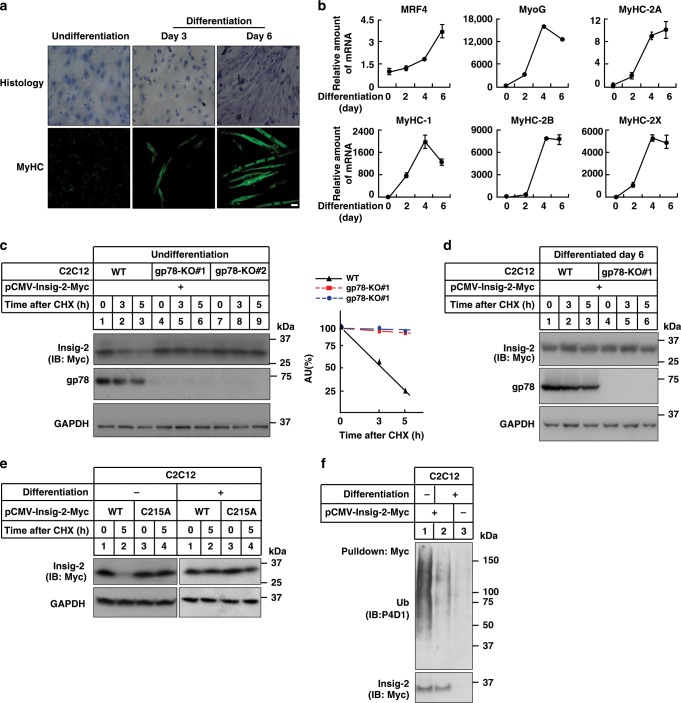
Gp78 mediates Insig-2 degradation in the undifferentiated myoblasts but not differentiated myotubes. **a** Histologic and anti-MyHC immunofluorescent images of undifferentiated and differentiated C2C12 cells. Scale bar, 20 μm. **b** Quantification of gene expression of several differentiation markers using RT-qPCR (*n* = 3 biologically independent samples per condition, bars in black). Data are presented as mean ± s.d. **c**, **d** Undifferentiated WT or two lines of *gp78*-knockout (*gp78*-KO#1 and #2) C2C12 cells (**c**) and WT or *gp78*-KO#1 C2C12 cells at Differentiation Day 6 (**d**) were transfected with a plasmid expressing Insig-2 and treated with 100 μM CHX for indicated time periods. Cells were harvested for immunoblotting analysis. Densitometric analysis of Insig-2 levels in (**c**). AU arbitrary unit. **e** Undifferentiated (−) and differentiated (+, Day 6) C2C12 cells were transfected with plasmids expressing WT or C215A mutant forms of Insig-2. Forty-eight hours later, cells were treated without or with 100 μM CHX for 5 h. Cells were harvested for immunoblotting analysis. **f** Undifferentiated (−) and differentiated (+) C2C12 cells were transfected with plasmids expressing Myc-tagged Insig-2 and HA-tagged ubiquitin. Forty-eight hours later, cells were treated with 10 μM MG132 for 5 h and harvested for ubiquitylation assay.

The interesting observation that *gp78* deficiency failed to elevate Insig-2 protein levels in mouse skeletal muscle (Fig. 1a) prompted us to investigate whether gp78 facilitates Insig-2 degradation in C2C12, an immortalized skeletal myoblast cell line derived from mouse muscle^20^. Consistent with the previous findings^21,22^, C2C12 cells remained undifferentiated in normal growth medium (containing 10% FBS) with fibroblast-like appearance and single rounded nucleus per cell (Fig. 3a). Under low serum (2% horse serum (HS)) conditions, however, C2C12 cells were induced to differentiate and fuse into elongated, multinucleated myotubes over time (Fig. 3a). Meanwhile, we detected drastically increased expression of differentiation markers including *Mrf4*, *MyoG*, *MyHC-2A*, *MyHC-1*, *MyHC-2B*, and *MyHC-2×* (Fig. 3b). These results validate our myoblast-to-myotube differentiation system.

We then generated two independent lines of *gp78*-deficient C2C12 cells (gp78-KO #1 and #2) using the CRISPR/Cas9 techniques and induced differentiation by supplementing cells with 2% HS. The CHX chase assay was performed to compare the half-life of transfected Insig-2 in undifferentiated and differentiated C2C12 cells. Similar to the observations in CRL1601 cells (Fig. 2a, lanes 1–3), Insig-2 was barely detectable at 5 h of CHX treatment in undifferentiated WT myoblasts (Fig. 3c, lanes 1–3). This Insig-2 degradation, however, was completely abrogated upon *gp78* deficiency (Fig. 3c, lanes 4–9). Insig-2 was very stable in both WT and *gp78* KO C2C12 cells 6 d after differentiation (Fig. 3d). The C215A mutation protected Insig-2 from degradation in the undifferentiated cells, with no obvious changes in the stability of WT and mutant Insig-2 proteins in the differentiated myotubes (Fig. 3e). Further, Insig-2 ubiquitylation was substantially reduced following differentiation (Fig. 3f). The immunoprecipitation (IP) assay revealed equal amounts of gp78 interacting with Insig-2 in both differentiated and undifferentiated cells (Supplementary Fig. 1g), suggesting that the binding of gp78 to Insig-2 in C2C12 cells is constitutive and independent of the differentiation state. We therefore concluded that gp78 accounts for ubiquitylation and degradation of Insig-2 in the undifferentiated but not differentiated C2C12 cells. These findings in the cultured cells are consistent with *gp78*^−*/−*^ mouse muscle results (Fig. 1a), both of which suggest that gp78-mediated Insig-2 ubiquitylation and degradation are blunted in the muscle.

### Cys215 of Insig-2 is oxidized by ROS

**Fig. 4:**
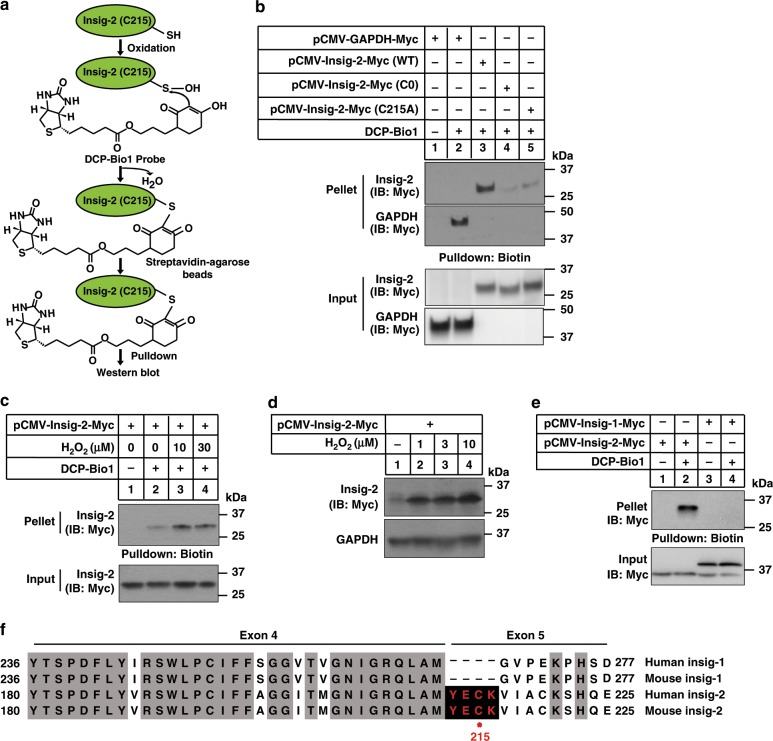
Cys215 of Insig-2 can be oxidized by ROS. **a** Procedure of detecting Cys215 oxidation in Insig-2 using the DCP-Bio1 probe. **b** The CRL1601 cells were transfected with indicated plasmids and harvested for cysteine sulfenic acid detection as described in the Materials and methods. **c** C2C12 cells were transfected with a plasmid expressing Insig-2 and then treated with H_2_O_2_ at indicated concentrations for 10 min. The cells were then harvested for cysteine sulfenic acid detection. **d** C2C12 cells were transfected with a plasmid expressing Insig-2 and then treated without (−) or with 10 μM H_2_O_2_ for 5 h. Cells were harvested for immunoblotting analysis. **e** CRL1601 cells were transfected with plasmids expressing Insig-1 or Insig-2 as indicated and then harvested for cysteine sulfenic acid detection. **f** Sequence alignment of human and mouse Insig-1 and Insig-2 proteins. Conserved sequences are highlighted in shadow. The YECK tetrapeptide (in red) is exclusively found in Insig-2 but not Insig-1 proteins. Red dot denotes the ubiquitylation site.

Since Cys215 is the ubiquitylation site of Insig-2 and cysteines can be readily oxidized, we took advantage of a cysteine sulfenic acid (-SOH) probe DCP-Bio1 to analyze the redox state of cysteine residues in Insig-2 protein (Fig. 4a). DCP-Bio1 recognizes the oxidized form of cysteines in vitro and in vivo^23^. GAPDH can be effectively detected by DCP-Bio1 (refs. ^24,25^) and was used as a positive control. The WT form of Insig-2 was successfully labeled by DCP-Bio1 whereas C0 and C215A mutations largely abolished probe recognition (Fig. 4b). These results suggest that Insig-2 is oxidizable and Cys215 is the major oxidation site. To determine whether Insig-2 stability is regulated by cellular redox, we transfected cells with Insig-2 followed by treatments with increasing concentrations of hydrogen peroxide (H_2_O_2_), an oxidant that can convert the cysteine residues to cysteine sulfenic acids^26,27^. H_2_O_2_ markedly increased Insig-2 oxidation (Fig. 4c) and protein levels (Fig. 4d). In addition, we also treated cells with three other ROS inducers (palmitic acid, pyocyanin, and menadione) and two scavengers (NAC and Trolox). The ROS levels were significantly increased by palmitic acid (PA), pyocyanin, and menadione, and decreased by NAC and Trolox (Supplementary Fig. 2a). Notably, the three ROS inducers elevated and the two ROS scavengers reduced Insig-2 level (Supplementary Fig. 2b). Importantly, Insig-1 was not labeled by the DCP-Bio1 probe (Fig. 4e). Sequence alignments showed that both human and mouse Insig-1 proteins lack the YEC_215_K tetrapeptide as found in Insig-2 (Fig. 4f). These data suggest Insig-2 stabilization is regulated by oxidation at the Cys215 residue.

### Ablation of *Insig-2* causes lipid accumulation

**Fig. 5:**
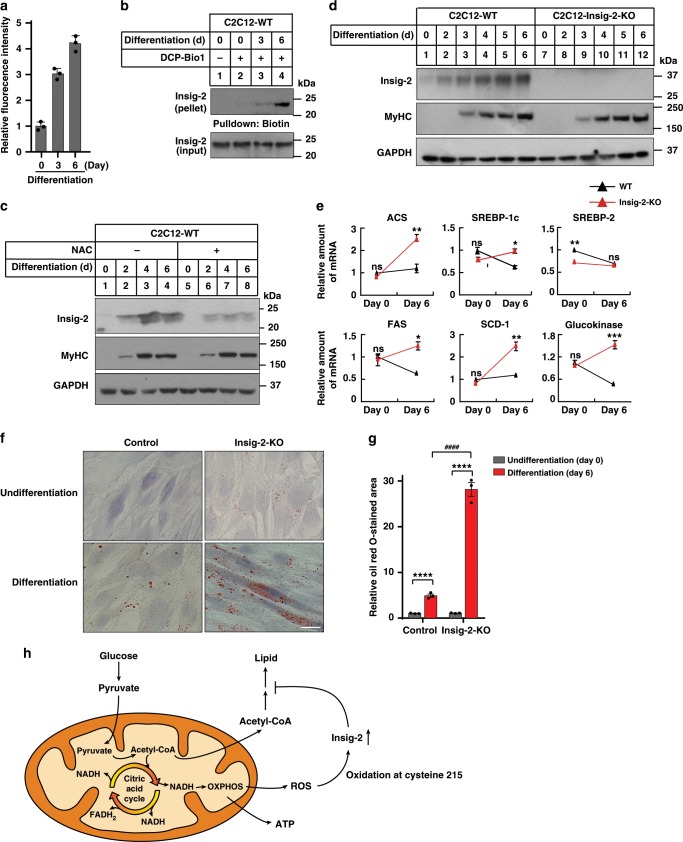
Depletion of Insig-2 increases lipid accumulation in differentiated C2C12 cells. **a** The ROS levels of C2C12 cells during differentiation as measured by dichlorofluorescein (DCF). Data are presented as mean ± s.d. (*n* = 3 independent experiments). **b** C2C12 cells were harvested for cysteine sulfenic acid detection at indicated differentiation days. **c** WT C2C12 cells treated without or with NAC at indicated differentiation days were harvested for immunoblotting analysis. **d** WT and *Insig-2*-KO C2C12 cells at indicated differentiation days were harvested for immunoblotting analysis. **e** The mRNA levels of indicated genes in the undifferentiated and differentiated C2C12 cells (*n* = 2 biologically independent samples per condition, bars in black). Data are presented as mean ± s.d. *p < 0.05, **p < 0.01, ***p < 0.001. ns not significant, two-way ANOVA followed by Bonferroni’s post hoc analysis. **f** Oil Red O staining, Scale bar, 20 μm. **g** Quantification of the lipid droplets of the cells in (**f**) (*n* = 3 biologically independent experiments). Data are presented as mean ± s.d. Asterisks indicate the differences of oil red O staining in WT (bars in black) or *Insig-2*-KO C2C12 cells (bars in red) between Differentiation Day 0 (undifferentiated) and 6, ****p < 0.0001; hash symbols indicate the differences of oil red O staining in WT and *Insig-2*-KO C2C12 cells at Differentiation Day 6, ^####^*p* < 0.0001, two-way ANOVA followed by Bonferroni’s post hoc analysis. **h** Schematic showing glucose metabolism in the skeleton muscle. The oxidation of C215 by ROS generated as side products during oxidative phosphorylation (OXPHOS) process stabilizes Insig-2, inhibiting lipid biosynthesis by blocking the SREBP pathway and helping to channel the carbon flux mainly to ATP generation.

We next investigated the mechanism by which Insig-2 is stabilized in the differentiated C2C12 cells. We measured the ROS level during myoblast differentiation and myotube formation. A gradual increase in the ROS level during C2C12 differentiation was revealed by dichlorofluorescein (DCF) (Fig. 5a). The ROS level in hepatic CRL1601 cells was in the middle (Supplementary Fig. 2c). Meanwhile, Insig-2 oxidation was increased over the differentiation period (Fig. 5b). This result supported our hypothesis that the skeletal muscle had higher ROS and Insig-2 was easier to be oxidized in skeletal muscle than in other tissues. In addition, we treated the WT C2C12 cells with the ROS scavenger NAC during differentiation. NAC did not affect the cell differentiation, as MyHC expression pattern was similar between control and NAC treatment. But the Insig-2 protein level in NAC-treated cells was much lower than in control cells (Fig. 5c). These results, together with those in Fig. 4, demonstrate an elevation in the cellular ROS level during muscle cell differentiation, which may lead to Insig-2 oxidation that competes with ubiquitylation-mediated degradation of the protein at Cys215.

To investigate the function of Insig-2 during C2C12 differentiation, we generated *Insig-2* KO cells using the CRISPR/Cas9 techniques. The expression pattern of MyHC (Fig. 5d) were similar between WT and Insig-2 KO cells during differentiation, indicating that Insig-2 is not required for myoblast differentiation. The protein level of Insig-2 was increased in WT cells over time (Fig. 5d), supporting the notion that differentiation elevates Insig-2 oxidation and thus prevents its ubiquitylation and degradation. Ablation of *Insig-2* significantly elevated the mRNA abundance of *Acs*, *Srebp-1c*, *Fas*, *Scd-1*, and *glucokinase* on differentiation day 6 (Fig. 5e), suggestive of enhanced lipid biosynthesis. The expression levels of *Adiponectin* were similar in WT and Insig-2 KO cells, indicating that Insig-2 deficiency does not interefere adipocyte differentiation (Supplementary Fig. 2d). Consistently, differentiated WT and *Insig-2* KO cells exhibited much more Oil Red O staining compared with undifferentiated counterparts, and ablation of *Insig-2* exacerbated the phenotype (Fig. 5f, g). In addition, we isolated satellite cells from WT and Insig-2 KO mice. The cells were induced to muscle cells with 2% HS for 2 days. The Insig-2-deficient primary muscle cells exhibited much more Oil Red O staining compared with WT cells (Supplementary Fig. 2e).

The skeleton muscle is the organ that consumes the majority of glucose in blood. In theory, glucose can be used to generate ATP through glycolysis, TCA cycle, and oxidative phosphorylation with ROS produced as side products. Acetyl-CoA derived from glucose can also be used to synthesize lipids through lipogenesis. The oxidation of C215 by ROS stabilizes Insig-2, thus blocking the SREBP pathway and lipid biosynthesis and helping to channel the carbon flux mainly towards ATP generation.

### Evolutionary conservation of *Insig-2* in amniotes

**Fig. 6:**
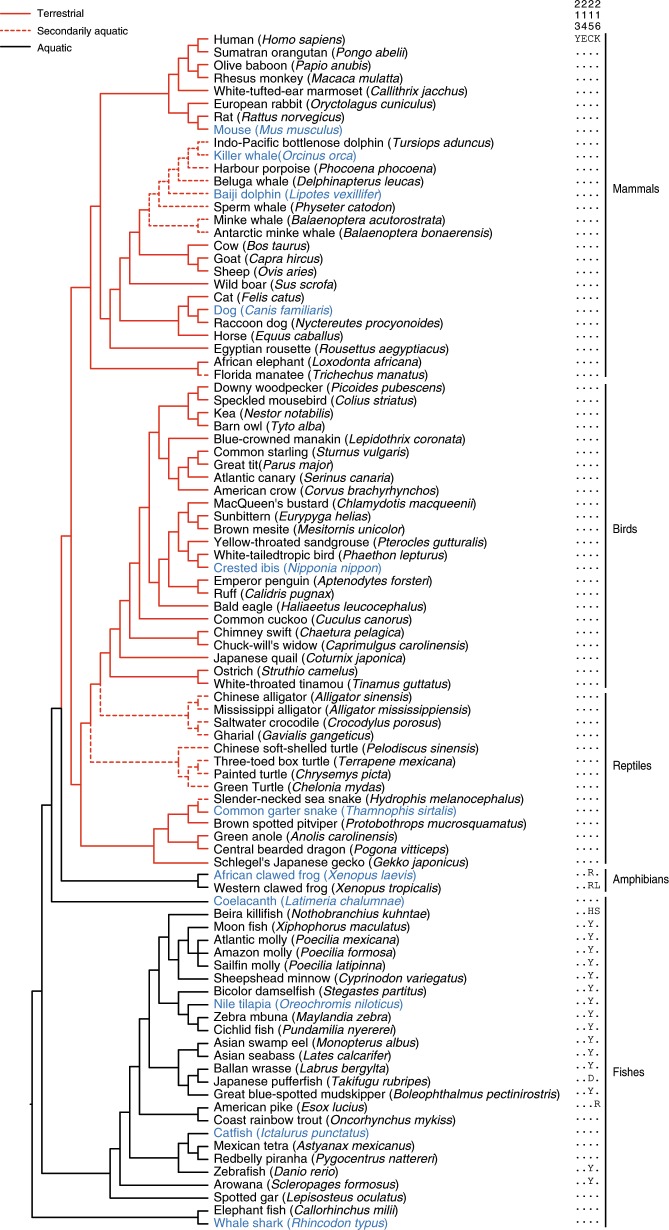
Sequence conservation of Cys215 of Insig-2 in amniotes during evolution. The tree shows the YECK tetrapeptide sequence (amino acids position of 213–216) of Insig-2 is highly conserved in amniotes (93 species in 5 categories, indicated by red lines), including secondarily aquatic cetaceans (indicated by red dashed lines), but not in aquatic amphibians and fishes (indicated by black lines). The species in blue were tested for their Insig-2 regulation by ROS in Fig. 7b, c.

To gain a broader picture of *Insig-2* evolution, we collected all available vertebrate *Insig* genes (Supplementary Data set 1). All accessions of genes and genomes are listed in Supplementary Table 1. We obtained complete coding sequences of *Insig-2* from 93 species of vertebrates in 5 major groups (mammals, birds, reptiles, amphibians, and fishes) (Fig. 6). By aligning deduced amino acid sequences of these genes, we found a highly conserved YECK tetrapeptide sequence in Insig-2 proteins from all examined reptiles, birds, and mammals (Fig. 6), including secondarily aquatic cetaceans who breathe air as their terrestrial ancestors did^28–30^. By contrast, most aquatic fishes and amphibians do not have the cysteine residue in such a tetrapeptide (Fig. 6). Based on the principle of parsimony, we speculate that all examined reptiles, birds and mammals have inherited the cysteine residue from their common ancestor of vertebrates, but most aquatic fishes and amphibians do not require such conservation of this residue.

We next examined whether differential selective pressures have acted on the *Insig-2* gene between the two vertebrate groups. By using a likelihood method, we estimated *ω*, the ratio of nonsyonymous to synonymous substitution rates, as a measure of evolutionary force. The *ω* values of less than, equal to, and more than 1 represent purifying selection, neutral evolution, and positive selection, respectively^31^. First, we assumed a same *ω* for the branches connecting all 93 vertebrates and estimated *ω* to be 0.053 (Model A in Supplementary Table 2). We tested whether a model that allows variable *ω* for each branch (Model B in Supplementary Table 2; see also Supplementary Fig. 3) fits the data significantly better than Model A. We found that Model B did have a significant better fit than Model A (*P* = 0.008, Supplementary Table 2), suggesting that heterogeneous selective pressures must have occurred along specific lineages of vertebrates. Second, we tested whether *ω* is on average lower or higher in amniotes than in aquatic fishes/amphibians. Given that secondarily aquatic vertebrates breathe air as their terrestrial ancestors did, we here treated these animals as amniotes. The two-ratio model (model B in Supplementary Table 2) assumes two *ω* values: branches in red have *ω*_1_ and branches in black have *ω*_0_ (Fig. 6), whereas the one-ratio model only estimates one uniform *ω*. Both *ω*_0_ (0.058) and *ω*_1_ (0.046) estimated by the two-ratio model were smaller than 1 (Supplementary Table 2), indicative of purifying selection. The model B was not significantly different from the model A (*P* = 0.062, Supplementary Table 2), suggesting that two groups of vertebrates have a similar *ω* value. Third, we tested the possibility of differential selective pressures between two groups of vertebrates by implementing Clade Model C (CmC) that includes 3 site classes. Classes 0 (*ω*_0_) and 1 (*ω*_1_) indicate purifying selection and neutral evolution, respectively, and are assumed to be shared between two groups, whereas the third site class is allowed to differ (*ω*_f_≠*ω*_2_) between the focal clade (amniotes, *ω*_f_) and the background (aquatic fishes/amphibians, *ω*_2_)^31^. After comparing with its null model (M2a_rel) that is same to CmC but assumes *ω*_f_ = *ω*_2_ (ref. ^32^), the difference between two models is statistically significant (*P* = 0.001, Supplementary Table 2), suggesting that the CmC model fits the data significantly better than its null model. A total of 15.3% amino acid sites of Insig-2 were identified to be under differential selection between amniotes and aquatic fishes/amphibians, with a lower *ω* value in the former (*ω*_f_ = 0.169) than the latter (*ω*_2_ = 0.283). The greater *ω* value suggests that *Insig-2* has been relaxed in aquatic vertebrates. Thus, functional divergence of *Insig-2* between amniotes and aquatic fishes/amphibians may have been shaped by differential selective pressures.

**Fig. 7:**
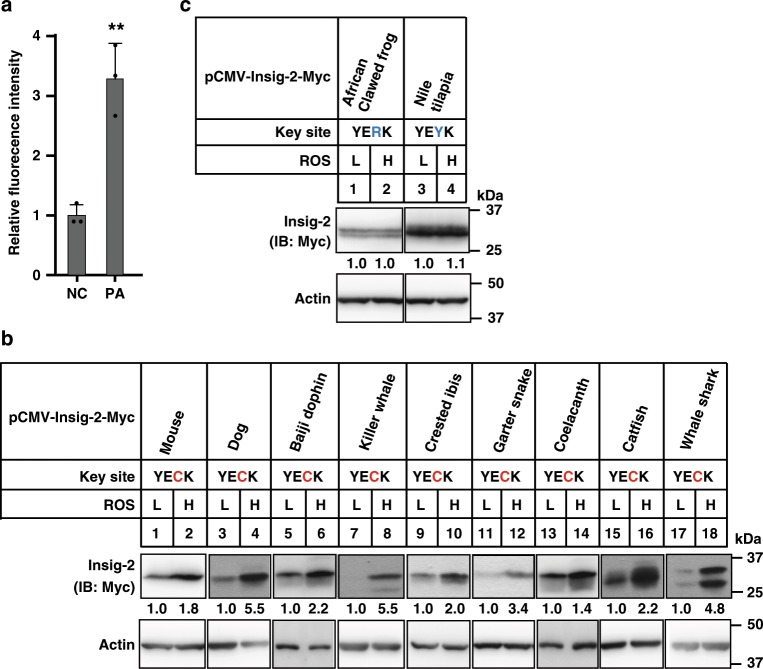
ROS induces stabilization of Insig-2 protein from representative species of amniotes. **a** The ROS levels of CRL1601 cells treated without (NC, negative control) or with 200 μM palmitic acid (PA) as measured by DCF. Data are presented as mean ± s.d. (*n* = 3 independent experiments). **p < 0.01, unpaired two-tailed Student’s *t* test. **b**, **c** CRL1601 cells were transfected with plasmids expressing Insig-2 from indicated species and treated without (L, low) or with 200 μM PA (H, high) for 16 h to induce ROS. Cells were harvested for immunoblotting analysis. Blots were quantified and the intensity of Insig-2 normalized to actin is indicated. Control treatment is defined as 1.

We next tested whether Insig-2 proteins containing the YECK tetrapeptide sequence are functionally conserved among amniotes. As shown earlier (Fig. 4), human Insig-2 protein is stabilized by oxidation on Cys215. To examine whether Insig-2 with YECK from other species can be regulated by ROS, we synthesized the *Insig-2* cDNAs of numerous species and cloned them into expression plasmids for the transfection experiments. We utilized PA to increase cellular ROS level (Fig. 7a) because this condition is more physiologically relevant than H_2_O_2_ treatment. Indeed, high ROS condition increased Insig-2 protein levels of mouse, dog, Baiji dolphin, killer whale, crested ibis, common garter snake, coelacanth, catfish, and whale shark (Fig. 7b). Those without YECK, as in African clawed frog and Nile tilapia, were not altered by ROS (Fig. 7c). Given that the Cys215 residue in Insig-2 is readily oxidized and that one of the major differences between water and land is oxygen concentration, we then looked into fishes that live in water but have equivalent Cys residues. Nine out of 29 fish species have YECK or YECR (Fig. 6), most of which can either breathe air with a primitive lung (e.g., coelacanth and spotted gar)^33^ or require higher concentration of oxygen in water to survive (e.g., catfish and coast rainbow trout)^34,35^. These exceptions in turn imply an important role of Cys215 oxidation during evolution.

We finally reconstructed the evolutionary history of *Insig-1* and *Insig-2* in vertebrates and invertebrates by retrieving genes from GenBank and identifying them from genome or transcriptome assemblies (Supplementary Table 1). Both *Insig-1* and *Insig-2* were identified in all examined vertebrates. By contrast, only one single gene (named *Insig-1/Insig-2*) was detected in all examined invertebrates, with the exception of purple sea urchin (Supplementary Fig. 4). The phylogenetic tree showed that vertebrate *Insig-1* and *Insig-2* formed two separate monophyletic groups deriving from invertebrate *Insig-1/Insig-2* (Supplementary Fig. 4), suggesting that the two duplicate genes may have resulted from the whole-genome duplication at the origin of vertebrates^36^.

## Discussion

In this study, we show that Cys215 is the key ubiquitylation site of human Insig-2, and it is also subjected to oxidation by ROS. During myoblast differentiation the cellular ROS level increases, which oxidizes Insig-2 on Cys215 and thus protects the protein from ubiquitylation-mediated degradation in proteasome. This oxidation-ubiquitylation competitive mechanism explains the muscle-specific stabilization of Insig-2 and prevents muscle cells from lipid overaccumulation.

The expression of Insig-1 and Insig-2 is differentially regulated at both transcriptional and posttranslational levels. Fasting induces high mRNA levels of *Insig-2* and low mRNA levels of *Insig-1* in the liver^37^. Upon refeeding, insulin downregulates *Insig-2a* mRNA, the major transcript of *Insig-2*, but upregulates the expression of *Insig-1* through activating the SREBP-1c pathway. At the protein level, Insig-1 is ubiquitylated and degraded rapidly in response to cholesterol depletion^9^. However, Insig-2 remains stable in cultured fibroblasts and CHO cells^38^ but is subjected to gp78-mediated ubiquitylation and degradation in mouse liver^14^. We here demonstrate that competitive oxidation and ubiquitylation on the cysteine 215 residue confers the tissue-specific stabilization of Insig-2, hereby reconciling the apparent discrepancy in previous studies. However, the oxidation of endogenous Insig-2 in muscle has not been directly illustrated. We cannot exclude the possibility that Insig-2 is stabilized by a different mechanism.

Insig-2 was stable in SRD-13A cells unless E214 was mutated to A^39^. The Insig-2(E214A) was ubiquitinated at K100 and K102 by the E3 gp78. However, the WT Insig-2 was ubiquitinated at C215 if it was not oxidized. The E214A mutation dramatically enhanced the Insig-2-gp78 interaction^40^. But the C215A did not affect Insig-2-gp78 interaction (Supplementary Fig. 1b). In addition, the ubiquitylation sites of Insig-2(WT) and Insig-2(E214A) were different, it was possible that gp78 bound WT and the E214A mutant Insig-2 in a different way.

The YECK tetrapeptide sequence is exclusively present in Insig-2 but not Insig-1. Evolutionary analysis suggests that the two *Insig* genes may have emerged at the origin of vertebrates through gene duplication (Supplementary Fig. 4). Interestingly, the coding sequence for YECK locates at the exon–intron junction (Fig. 4f), suggesting that alternative splicing may account for the appearance/disappearance of YECK in Insig-2/Insig-1. The Insig-2 proteins in mammals, birds, and reptiles contain the conserved YECK sequence, whereas Insig-2s in most of aquatic fishes and amphibians do not. ROS can stabilize YECK-containing Insig-2 proteins from several distinct species (Fig. 7b), but is ineffective on Insig-2 proteins lacking the YECK sequence (Fig. 7c).

We searched human database in http://myhits.vital-it.ch/cgi-bin/pattern_search and found about 148 proteins with YECK motif. We randomly chose three membrane proteins to test whether the Cys can affect protein stability. Their half-lives were not affected by the C to A mutation (Supplementary Fig. 5). These results suggest that YECK is necessary, but not sufficient for the ROS-regulated protein stability.

Why is Insig-2 selectively stabilized in muscle? The skeletal muscle is the major glucose-consuming tissue that takes up approximately 80% of glucose in the postprandial state^41^. In addition, glucose uptake in exercising skeletal muscle is at least six times more than resting skeletal muscle^42^. Glucose can be used to generate metabolic energy with ROS as side products^43^, as well as to be converted to fatty acids through lipogenesis pathway that is negatively controlled by Insigs. Therefore, our data suggest that the ROS burden may be higher in muscle than the other detected tissues, which is the major reason accounting for the tissue-selectivity of Insig-2 stablization. Considering the conservation of the YECK tetrapeptide of Insig-2 in amniotes but not in aquatic animals, we hypothesize that such conservation is not required in aquatic vertebrates due to the low concentration of oxygen. The elevated ROS in muscle induces Insig-2 oxidization at Cys215, blocks ubiquitylation and degradation of the protein, which inhibits lipid biosynthesis through attenuating the SREBP pathway and accelerating degradation of HMGCR. This stabilization of Insig-2 by oxidation prevents muscle cells from lipid overaccumulation and could assure proper muscle function that is essential for amniotes during predator-prey interaction. However, for most aquatic vertebrates, the oxygen content in water is much lower and oxidative stress is less^44^, correlating with the absence of the cysteine residue in the corresponding tetrapeptide. As the vertebrates transitioned to terrestrial environments about 380 million years ago when atmospheric O_2_ was about 25% and rapidly rose to >30%^45–49^ (Supplementary Fig. 6). It will be interesting to investigate whether the ROS-regulated stabilization of Insig-2 plays a role in water-to-land transition.

The oxidation-ubiquitylation competition has been previously reported occurred on acyl-CoA: cholesterol acyltransferase-2 (ACAT-2) protein^18^. The ubiquitylation site of ACAT-2 is also a cysteine residue (Cys277) and can be oxidized by lipid-induced ROS. The oxidation of Cys277 stabilizes ACAT-2, which then converts excess cholesterol and fatty acids to cholesteryl esters so as to reduce lipotoxicity. Here, ROS can competitively block ubiquitylation of Insig-2 on the Cys215 residue via oxidization, stabilizing the protein, and preventing lipid accumulation in muscles. Therefore, competitive oxidation and ubiquitylation on cysteine may be a common mechanism regulating lipid metabolism in response to oxidative stress.

## Methods

### Reagents

We obtained cycloheximide, diethylene triamine pentacetate acid (DTPA), N-ethylmaleimide (NEM), iodoacetamide, catalase, palmitic acid, fatty acid-free bovine serum albumin (BSA), protein inhibitors, 2′, 7′-Dichlorofluorescein diacetate (DCFH-DA) fluorescent dyes, Oil Red O, anti-Myc-coupled agarose beads from Sigma; high capacity NeutrAvidin agarose, mini dialysis devices, 10K MWCO, and ECL from ThermoFisher Scientific. We obtained Ni-NTA agarose beads from Qiagen; MG132 from Calbiochem; M-MLV Reverse Transcriptase from Promega; KOD Hot Start DNA Polymerase from Novagen; Taq polymerase, dNTPs, and X-Gal from TaKaRa; Quick change Site-Directed Mutagenesis kit from Stratagene; Cysteine Sulfenic acid probe DCP-Bio1 from Millipore; hydrogen peroxide (H_2_O_2_) from Sinopharm Chemical Reagent Co.,Ltd.; hematoxylin from Beyotime.

### Primary antibodies

Primary antibodies used for immunoblotting were as follows: mouse monoclonal anti-ubiquitin antibody P4D1 (SC-8017, Santa Cruz, 1:500); mouse monoclonal anti-β-actin (A1978, Sigma, 1:10,000); rabbit polyclonal anti-Myc (06-549, Millipore, 1:1000); rabbit polyclonal anti-Insig-2, rabbit polyclonal anti-gp78, and mouse monoclonal anti-Myc (9E10) were prepared in our laboratory.

### Plasmids

The expression plasmids of 5× Myc-Insig-2, 5× Myc-Insig-1, 5× Myc-GAPDH, HA-Ub, His_6_-Ub, and HA-gp78 were constructed by standard molecular cloning techniques. Various Insig-2 site-directed mutants were constructed using the QuikChange site-directed kits and confirmed by sequencing. The *Insig-2* cDNAs of representative species were synthesized and cloned into expression plasmid pCMV-5× Myc by Nanjing IDO biotechnology company.

### Animals and treatment

The *gp78*^*f/f*^ mice were crossed with cytomegalovirus (CMV) promoter-driven Cre mice (B6.C-Tg(CMV-cre)1Cgn/J, JAX 006054) to generate whole-body *gp78* knockout mice (*gp78*^*−/−*^) and crossed with C57BL/6J background for more than ten generations. In addition, *Insig1*^*f/f*^ *Insig2*^*−/−*^ (B6;129S6-*Insig1*^*tm1Mbjg*^ *Insig2*^*tm1Mbjg*^/J, JAX 005939) mice were crossed with C57BL/6J mice to generate global *Insig2* knockout mice (*Insig2*^*−/*−^), which were then backcrossed to the C57BL/6J mice for more than ten generations. Mice were housed under a 12-h light/dark cycle in a pathogen-free, temperature controlled room. Eight-week-old male mice were treated as indicated in the figure legends. All animal experiments were approved and supervised by the Laboratory Animal Ethical Committee of Wuhan University.

### Media

Medium A contains Dulbecco’s Modified Eagle Medium (DMEM) containing 100 units mL^−1^ penicillin and 100 μg mL^−1^ streptomycin sulfate. Medium B contains a 1:1 mixture of Ham’s F-12 Medium and DMEM containing 100 units mL^−1^ penicillin and 100 μg mL^−1^ streptomycin sulfate. Lipid-depleting medium was prepared by adding 5% lipoprotein-deficient serum, 1 μM lovastatin and 50 μM mevalonate into each medium.

### Cell culture

CHO-7 (CHO cell, a clone of CHO-K1 cells) was a generous gift from Dr. Joseph Goldstein at UT Southwestern Medical Center, USA. CRL1601 (McArdle RH7777, rat liver cells) and C2C12 (mouse skeletal muscle cells) were purchased from ATCC. All cells were grown in a monolayer at 37 °C with 5% CO_2_. CRL1601 cells were maintained in medium A supplemented with 10% fetal bovine serum (FBS). CHO-7 cells were maintained in medium B supplemented with 5% FBS. C2C12 cells were maintained in medium A supplemented with 10% FBS. C2C12-sgRNAs-gp78, C2C12-sgRNAs-Insig-2 cells were generated by the CRISPR-Cas9 technology and maintained in medium A supplemented with 10% FBS. C2C12 cells were induced to differentiate in medium A supplemented with 2% HS. Muscle stem cells were grown in F10 medium with 20% FBS, plus IL-1α, IL-13, TNF-α, IFN-γ, and bFGF.

### Transfection

C2C12 cells were transfected with indicated plasmids using lipofectamine 3000 transfection reagent (ThermoFisher Scientific). Other cells were transfected using Fugene HD (Promega) according to the manufacturer’s instructions.

### Measurement of intracellular ROS levels

Intracellular ROS level was determined using oxidation-sensitive DCFH-DA fluorescent dyes. Cells were washed twice with PBS and labeled on the culture plates with DCFH-DA for 30 min at 37 °C in serum-free medium. At the end of incubation, culture plates were trypsinized, resuspended in PBS and analyzed using a FACScan flow cytometer (*λ*_ex_ = 488 nm and *λ*_em_ 530 nm band-pass filter). The mean fluorescence intensity of 10,000 cells was measured in each sample and corrected for autofluorescence from unlabeled cells.

### Detection of cysteine sulfenic acid by DCP-Bio1 probe

Cysteine sulfenic acid detection by DCP-Bio1 probe was performed^50^. The treated cells were washed with PBS and homogenized in DCP-Bio1 working lysis buffer (1× PBS, 1% NP40, 1% sodium deoxycholate, 5 mM EDTA, 5 mM EGTA, 1 mM PMSF, 0.1 mM aprotinin, 0.1 mM leupeptin, 10 mM NEM, 10 mM iodoacetamide, 100 µM DTPA, and 200 U mL^−1^ catalase). Lysates were centrifuged at 12,000 × *g* for 10 min. Supernatants were collected and incubated with 1 mM DCP-Bio1 probe for 2 h on ice. For affinity capture of the labeled proteins, unreacted DCP-Bio1 was removed using a dialysis device. Dialyzed Samples were precleared with Sepharose CL-4B beads (Sigma), applied to plugged columns containing high-capacity streptavidin-agarose beads (ThermoFisher Scientific) and incubated overnight at 4 °C. Beads were then subjected to a series of stringent washes (at least four column volumes and two washes each) using 1% sodium dodecyl sulfate (SDS), 4 M urea, 1 M NaCl, 10 mM DTT, 100 μM ammonium bicarbonate and water. Beads were boiled for 10 min and samples were analyzed by immunoblotting analysis.

### RNA interference

Duplexes of siRNA targeting rat *gp78* (5′-CAUGCAGAAUGUCUCUUAAdTdT-3′) were synthesized by Genepharma (Shanghai, China). Transfection of siRNA was carried out using lipofectamine RNAiMAX reagent (ThermoFisher Scientific).

### Ubiquitylation assays

For ubiquitylation assay in the non-denaturing condition, cells were homogenized in buffer A (1× PBS, 1% NP-40, 1% sodium deoxycholate and 5 mM EDTA, 5 mM EGTA, 0.1 mM leupeptin) supplemented with protease inhibitors (10 μM MG132, 10 mM NEM, and cocktail). Immunoprecipitation was carried out with the anti-Myc agarose beads. For ubiquitylation assay in the denaturing conditions, cells were homogenized in 0.1 mL buffer A supplemented with protease inhibitors (10 μM MG132, 10 mM NEM, and cocktail). After sonication and centrifugation, supernatants were collected and mixed with 0.3 mL 8 M urea, rotated for 10 min at room temperature, diluted with buffer A to make final concentration of urea to 2 M, and subjected to IP with the anti-Myc agarose. Beads were boiled for 10 min or incubated with the Myc peptide for 3 h at 4 °C, and supernatants were analyzed by immunoblotting with indicated antibodies. No DTT or BME was applied unless indicated.

### Coimmunoprecipitation

Cells were harvested and lysed in 0.6 mL of IP buffer (1× PBS, 1% digitonin, 5 mM EDTA, 5 mM EGTA, and cocktail) followed by centrifugation at 12,000 × *g* for 10 min at 4 °C. IP was carried out with indicated antibody-coupled agarose, washed with IP buffers followed by western blotting^6^.

### Immunoblotting analysis

Cells were harvested and homogenized in 120 μL of RIPA buffer supplemented with protease inhibitors. Protein concentration of whole cell lysates was determined according to the Lowry method (Bio-Rad). Samples were mixed with 4× SDS loading buffer (150 mM Tris-HCl, pH 6.8, 12% SDS, 30% glycerol, 0.02% bromophenol blue, 6% BME) and boiled for 10 min. Proteins were resolved by SDS polyacrylamide gel electrophoresis and transferred onto polyvinylidene fluoride membranes. Immunoblots were blocked with 5% milk in TBST containing 0.075% Tween and probed with primary antibodies overnight at 4 °C. After washing in TBST 3 times, blots were incubated with secondary antibodies for 1 h at room temperature^51^.

### Immunofluorescence

Cells grown on coverslips were fixed with 4% paraformaldehyde for 30 min and incubated with 3% BSA in PBS containing primary antibodies (1:500) for 1 h at RT. After washing 3 times with PBS, cells were incubated with PBS containing 3% BSA and corresponding secondary antibodies (1:500) for 1 h at RT^52^.

### RT (reverse transcription)-qPCR

Total RNA was extracted using Trizol reagents (ThermoFisher Scientific) and subjected to RT with oligo dT followed by qPCR using target-specific primers in the Stratagene Mx30005P Q-PCR Systems. All reactions were prepared in triplicate and the relative amounts of mRNAs were calculated using the comparative CT method. Mouse *GAPDH* was used as controls. Values show the amount of mRNA relative to control sample, which is arbitrarily defined as 1. Primer sequences are used as follows:

Mouse *Insig-2* (Forward: 5′-CGGAAGATGCTGGAACCTGA-3′, Reverse: 5′-TGTGCTCTCCATACGCTCTCC-3′);

Mouse *gp78*: (Forward: 5′-CTGCCCTGTGGACATCTTTT-3′, Reverse: 5′-GACCCATCGAAGTGGAAGAA-3′);

Mouse *SREBP-1a*: (Forward: 5′-GGCCGAGATGTGCGAACT-3′, Reverse: 5′-TTGTTGATGAGCTGGAGCATGT-3′);

Mouse *SREBP-1c*: (Forward: 5′-GGAGCCATGGATTGCACATT-3′, Reverse: 5′-GGCCCGGGAAGTCACTGT-3′);

Mouse *SREBP-2*: (Forward: 5′-GCGTTCTGGAGACCATGGA-3′, Reverse: 5′-ACAAAGTTGCTCTGAAAACAAATCA-3′);

Mouse *Insig-1*: (Forward: 5′-TCACAGTGACTGAGCTTCAGCA-3′, Reverse: 5′-TCATCTTCATCACACCCAGGAC-3′);

Mouse *HMGCR*: (Forward: 5′-CTTGTGGAATGCCTTGTGATTG-3′, Reverse: 5′-AGCCGAAGCAGCACATGAT-3′);

Mouse *FDS:* (Forward: 5′-ATGGAGATGGGCGAGTTCTTC-3′, Reverse: 5′-CCGACCTTTCCCGTCACA-3′);

Mouse *FAS*: (Forward: 5′-GCTGCGGAAACTTCAGGAAAT-3′, Reverse: 5′-AGAGACGTGTCACTCCTGGACTT-3′);

Mouse *ACC*: (Forward: 5′-TGACAGACTGATCGCAGAGAAAG-3′, Reverse: 5′-TGGAGAGCCCCACACACA-3′);

Mouse *SCD-1*: (Forward: 5′-CCGGAGACCCCTTAGATCGA-3′, Reverse: 5′-TAGCCTGTAAAAGATTTCTGCAAACC-3′);

Mouse *GPAT*: (Forward: 5′-CAACACCATCCCCGACATC-3′, Reverse: 5′-GTGACCTTCGATTATGCGATCA-3′);

Mouse *ME*: (Forward: 5′-GCCGGCTCTATCCTCCTTTG-3′, Reverse: 5′-TTTGTATGCATCTTGCACAATCTTT-3′);

Mouse *G6PD*: (Forward: 5′-GAACGCAAAGCTGAAGTGAGACT-3′, Reverse: 5′-TCATTACGCTTGCACTGTTGGT-3′).

Rat *UBE2G2*: (Forward: 5′-TGGGAGGCATTGATCATGGG-3′, Reverse: 5′-GCAGGATGGAGATGCACACT-3′).

### Evolutionary analysis

We collected all available vertebrate *Insig-2* genes from the National Center for Biotechnology Information (NCBI), and employed TBLASTN^53^ using the human Insig-2 sequence as query to identify cetacean *Insig-2* genes in six cetacean genomes and one reptile genome. Complete coding sequences of *Insig-2* from 86 species of vertebrates were downloaded from GenBank (NCBI, www.ncbi.nlm.nih.gov). *Insig-2* genes from six species of cetaceans (Indo-Pacific bottlenose dolphin, harbor porpoise, beluga whale, sperm whale, minke whale, and Antarctic minke whale) and one species of reptile (slender-necked sea snake) were identified from their genome assemblies by TBLASTN searches^53^. We also conducted TBLASTN searches to identify *Insig-2* genes from invertebrate genome and transcriptome assemblies, using Insig-2 protein sequences from human, western clawed frog, zebrafish, elephant shark, and Mississippi alligator as queries. Protein sequences of Insig-2 were aligned by the MUSCLE program^54^ to examine the presence of YECK tetrapeptide sequence. Tests of differential selection were conducted by the CODEML program in PAML 4.7^31^. Likelihood ratio tests were applied to compare the difference between two nested models^31^. The input tree of vertebrates was obtained from TimeTree (www.timetree.org).

Complete coding sequences of *Insig-1* of 6 representative species of vertebrates (human, Mississippi alligator, chicken, zebrafish, western clawed frog, and elephant fish) were downloaded from GenBank. We used protein sequences of the 6 *Insig-1* genes to perform TBLASTN searches against genome and transcriptome assemblies from 9 representative species of invertebrates (purple sea urchin, Pacific oyster, giant owl limpet, California two-spot octopus, sea walnut, starlet sea anemone, lamp shell, blood fluke, and sponge). In case that we found a full-length gene in a transcriptome assembly, we used this sequence as query to conduct TBLASTN searches against the corresponding genome assembly, with an aim of detecting another potential copy of this gene. Except for purple sea urchin, all remaining eight species of invertebrates examined in this study only have one copy of the *Insig* gene, which we named as *Insig-1/Insig-2* in invertebrates. Both *Insig-1* and *Insig-2* genes from 6 vertebrates and *Insig-1/Insig-2* genes from nine invertebrates were aligned with the MUSCLE program. Following protein sequence alignment, the nucleotide sequence alignment was generated and subsequently used to build a phylogenetic tree^55^. After selecting the best-fitting model of nucleotide substitution by ModelTest^56^, the phylogenetic tree was reconstructed by the Bayesian approach using MrBayes v3.1.2 (ref. ^57^).

### Statistical analyses and reproducibility

All statistical analyses were performed using the Graphpad Prism. Data were expressed as means ± s.d. and analyzed by unpaired two-tailed Student’s *t* test or two-way ANOVA followed by Bonferroni’s post hoc analysis as indicated. Statistical significance was set at *p* < 0.05. Sample sizes, statistical tests and *p* values for each experiment are depicted in relevant figure legends.

### Reporting summary

Further information on research design is available in the Nature Research Reporting Summary linked to this article.

## Supplementary information

Supplementary Information

Description of Additional Supplementary Files

Supplementary Data 1

Reporting Summary

## Data Availability

The data that support the findings of this study are available within the paper and its Supplementary Information files, and from the corresponding author upon reasonable request.

## Source Data

Source Data
