# Supplementary material for: Competitive oxidation and ubiquitylation on the evolutionarily conserved cysteine confer tissue-specific stabilization of Insig-2
Source: Nat Commun. 2020 Jan 17;11:379. doi: 10.1038/s41467-019-14231-w (PMC6969111; doi:10.1038/s41467-019-14231-w)
Supplement: Supplementary file 1 — Supplementary Information [file 41467_2019_14231_MOESM1_ESM.pdf]

Supplementary Information

Competitive oxidation and ubiquitylation on the evolutionarily conserved cysteine confer tissue-specific stabilization of Insig-2

Zhou *et al.*

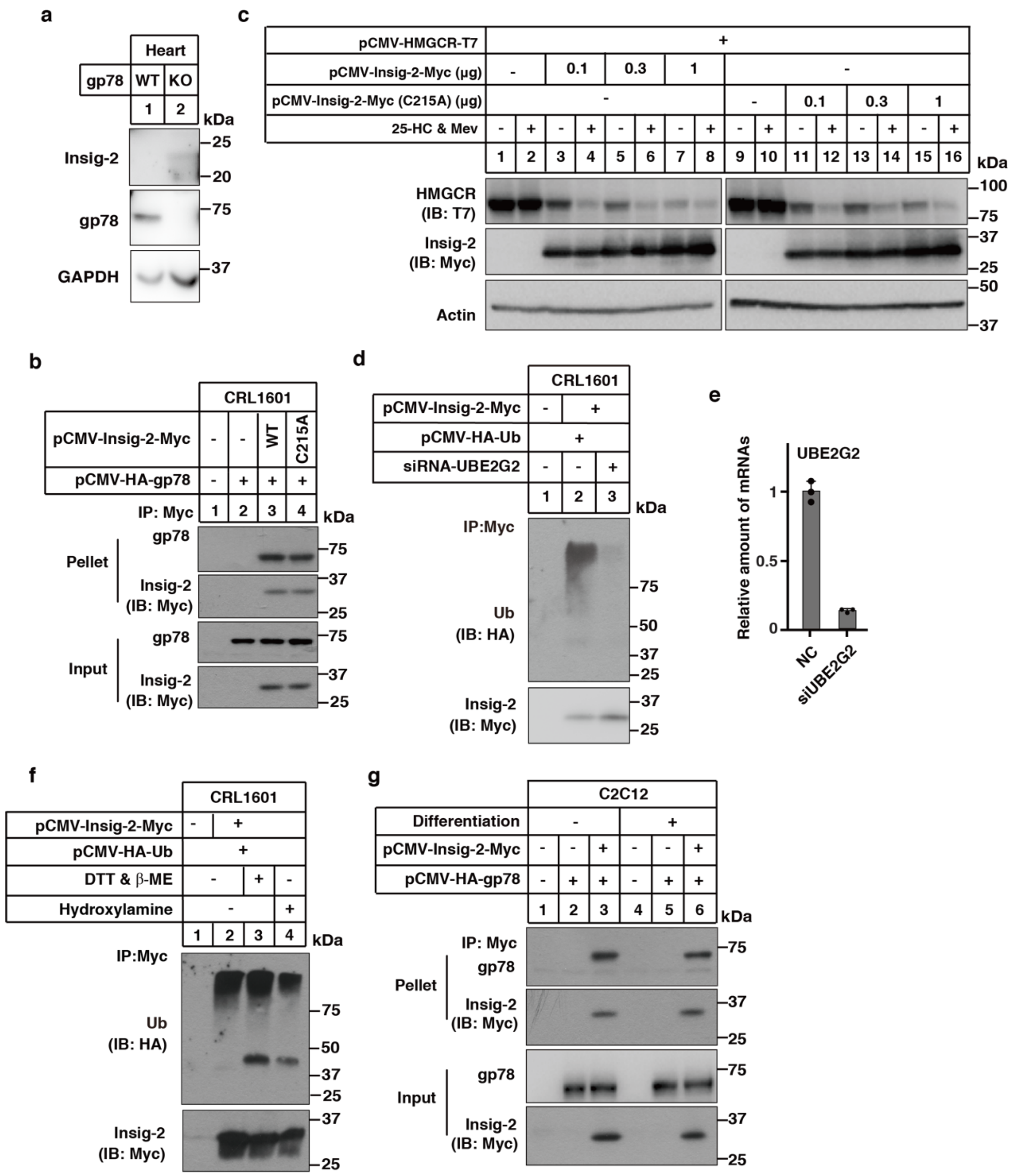

Supplementary Figure 1. Characterization of Insig-2.

(a) Expression of indicated proteins in heart of WT and gp78 KO mice. (b) CRL1601 cells were transfected with indicated plasmids. Cells were then harvested for co-immunoprecipitation (IP) analysis with the anti-Myc coupled agarose beads. (c) The cells were transfected with indicated plasmids, depleted of lipids and treated with 25-HC (1 μg/ml) and mevalonate (10 mM) for 5 h. Cells were harvested for immunoblotting analysis. (d) CRL1601 cells were transfected with indicated plasmids

and siRNAs, and treated with 10 μM MG132 for 5 h. Cells were harvested for ubiquitylation assay. (e) Knock-down efficiency of UBE2G2 (n=3 biologically independent samples per condition, bars in black). (f) CRL1601 cells were transfected with indicated plasmids and treated with 10 μM MG132 for 5 h. Cells were harvested, lysed in denaturing IP buffer followed by incubation with the anti-Myc beads. Precipitates were treated with (+) or without (-) DTT (75 mM) and β-ME (1.5%) or 0.5 M hydroxyl amine prior to immunoblotting analysis. (g) Undifferentiated (-) and differentiated (+) C2C12 cells were transfected with indicated plasmids and then harvested for co-IP analysis with the anti-Myc coupled agarose beads.

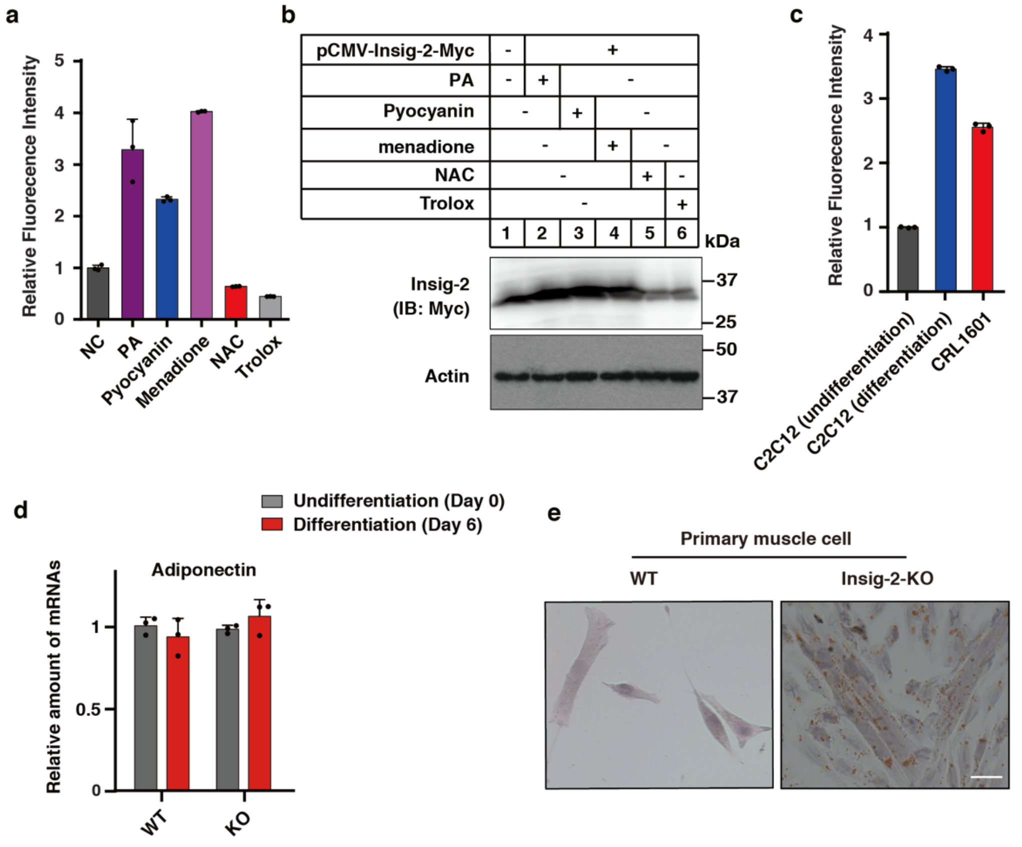

Supplementary Figure 2. Insig-2 can be oxidized and stabilized by ROS and it prevents lipid accumulation in primary muscle cells.

(a) The ROS levels of cells treated with three ROS inducers (PA, pyocyanin and menadione) and two scavengers (NAC and Trolox) as measured by dichlorofluorescein (DCF) (n=3 biologically independent samples per condition, bars in black). (b) Cells were treated with three ROS inducers (PA, pyocyanin and menadione) and two scavengers (NAC and Trolox). Then cells were harvested for immunoblotting analysis. (c) The ROS levels of CRL1601, undifferentiated and differentiated C2C12 cells as measured by DCF. Data are presented as mean ± s.d. (n = 3 independent experiments). (d) The mRNA levels of Adiponectin in undifferentiated and differentiated WT and Insig-2 knock out cells (n=3 biologically independent samples per condition, bars in black). (e) Oil Red O staining of primary muscle cells from WT and *Insig-2*-KO mice. Scale bars, 20 μm.

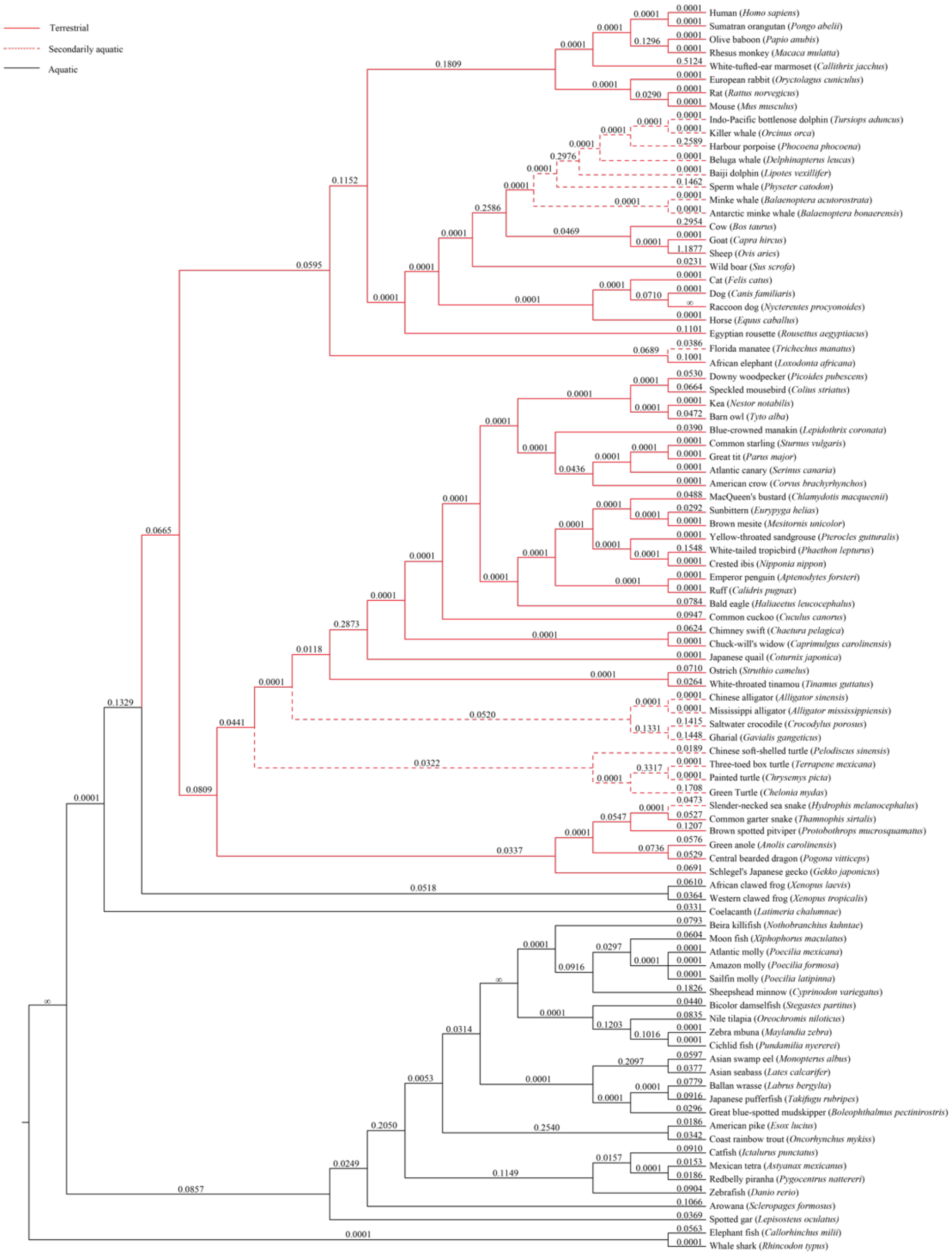

Supplementary Figure 3. The Bayesian tree of Insig genes in animals showing the duplication event at the origin of vertebrates. Branch lengths are drawn to the scale, numbers at the nodes are Bayesian posterior probabilities as percentages.

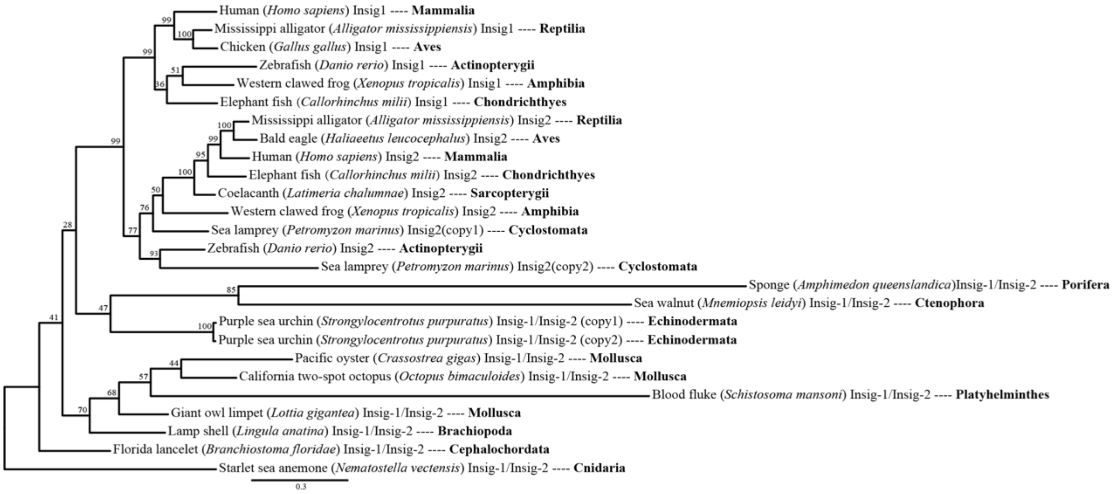

Supplementary Figure 4. The results of the free-ratio model. The phylogeny used here is the same as in the figure 6. ω value for each branch was mapped to the phylogeny. ∞ represents ds=0 but dn≠0.

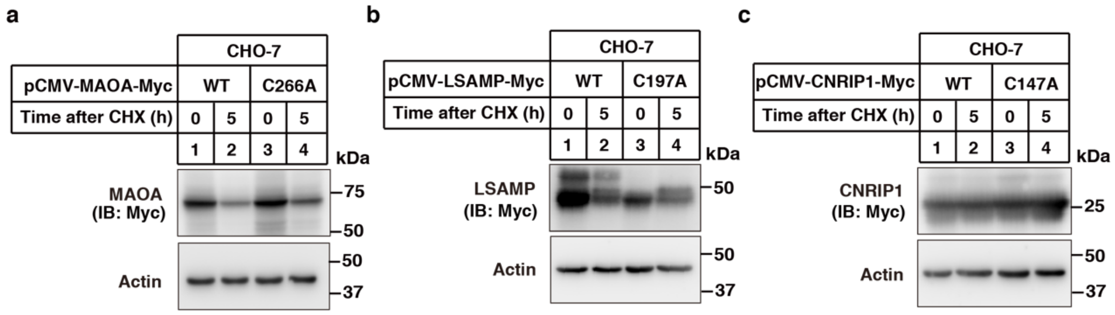

Supplementary Figure 5. The cysteine of the YECK motif in MAOA, LSAMP and CNRIP1 did not affect their protein stability.

(a-c) The Monoamine Oxidase A (MAOA), Limbic System Associated Membrane Protein (LSAMP) and Cannabinoid Receptor Interacting Protein 1 (CNRIP1) proteins have YECK motif. We made the expression plasmids encoding them or their C-to-A mutant proteins. CHO-7 cells were transfected with plasmids expressing the indicated proteins, and then treated without or with 100 µM CHX for 5 h followed by immunoblotting analysis.

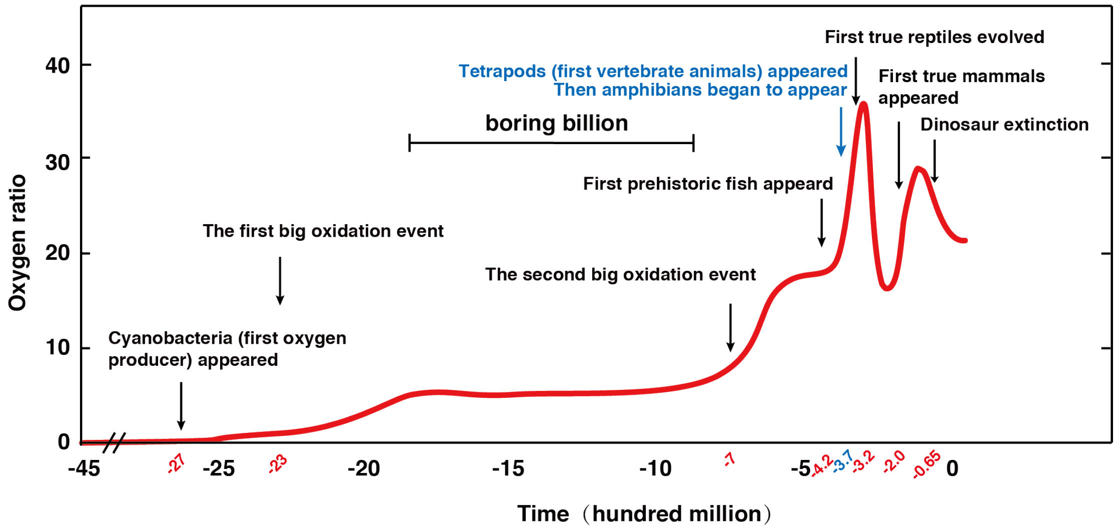

Supplementary Figure 6. The atmospheric O_2 concentration and major evolutionary events from the ancient time to the present.

Supplementary Table 1. GenBank accession numbers of *Insig-1* and *Insig-2* genes and information of genome and transcriptome assemblies at NCBI used for identifying the two genes.

**Accession numbers for vertebrate *Insig-2* genes**			
**Common name**	**Species name**	**Class**	**Accession No.**
Human	*Homo sapiens*	Mammalia	NM_001321329.1
Olive baboon	*Papio anubis*		NM_001168911.1
Rhesus monkey	*Macaca mulatta*		XM_002799405.2
White-tufted-ear marmoset	*Callithrix jacchus*		XM_017973529.1
Dog	*Canis familiaris*		XM_848012.5
African elephant	*Loxodonta africana*		XM_010596395.2
Mouse	*Mus musculus*		NM_001357251.1
Baiji dolphin	*Lipotes vexillifer*		XM_007456350.1
Killer whale	*Orcinus orca*		XM_004276497.2
Goat	*Capra hircus*		NM_001285738.1
Sheep	*Ovis aries*		XM_004004747.3
Cow	*Bos taurus*		XM_024981026.1
Raccoon dog	*Nyctereutes procyonoides*		HQ878328.1
Egyptian rousette	*Rousettus aegyptiacus*		XM_016151389.1
Cat	*Felis catus*		XM_003990710.5
Horse	*Equus caballus*		XM_001488083.5
Sumatran orangutan	*Pongo abelii*		XM_024243812.1
Wild boar	*Sus scrofa*		NM_001129968.1
Rat	*Rattus norvegicus*		BC085682.1
European rabbit	*Oryctolagus cuniculus*		XM_002712385.3
Florida manatee	*Trichechus manatus latirostris*		XM_004389679.2

Ostrich	*Struthio camelus*	Aves	XM_009677191.1
White-throated tinamou	*Tinamus guttatus*		XM_010211730.1
Japanese quail	*Coturnix japonica*		XM_015868738.1
Ruff	*Calidris pugnax*		XM_014939905.1
Emperor penguin	*Aptenodytes forsteri*		XM_009290976.2
American crow	*Corvus brachyrhynchos*		XM_008638221.2
Great tit	*Parus major*		XR_001522851.2
Atlantic canary	*Serinus canaria*		XM_009087804.2
Common starling	*Sturnus vulgaris*		XM_014871832.1
Common cuckoo	*Cuculus canorus*		XM_009571512.1
Yellow-throated sandgrouse	*Pterocles gutturalis*		XM_010080588.1
Chimney swift	*Chaetura pelagica*		XM_010001284.1
Brown mesite	*Mesitornis unicolor*		XM_010182157.1
Kea	*Nestor notabilis*		XM_010020908.1
Crested ibis	*Nipponia nippon*		XM_009461760.1
Chuck-will's widow	*Caprimulgus carolinensis*		XM_010176721.1
Barn owl	*Tyto alba*		XM_009969438.1
Sunbittern	*Eurypyga helias*		XM_010150819.1
Blue-crowned manakin	*Lepidothrix coronata*		XM_017820922.1
Bald eagle	*Haliaeetus leucocephalus*		XM_010575124.1
White-tailed tropicbird	*Phaethon lepturus*		XM_010290335.1
MacQueen's bustard	*Chlamydotis macqueenii*		XM_010120675.1
Downy woodpecker	*Picoides pubescens*		XM_009904412.1
Speckled mousebird	*Colius striatus*		XM_010204276.1
Central bearded dragon	*Pogona vitticeps*	Reptilia	XM_020779611.1
Schlegel's Japanese gecko	*Gekko japonicus*		XM_015406308.1

Green anole	*Anolis carolinensis*		XM_008109701.2
Mississippi alligator	*Alligator mississippiensis*		XM_019480585.1
Chinese alligator	*Alligator sinensis*		XM_006017890.3
Brown spotted pitviper	*Protobothrops mucrosquamatus*		XM_015820283.1
Common garter snake	*Thamnophis sirtalis*		XM_014055045.1
Three-toed box turtle	*Terrapene mexicana*		XM_024224508.1
Green Turtle	*Chelonia mydas*		XM_007057044.1
Chinese soft-shelled turtle	*Pelodiscus sinensis*		XM_006137623.2
Painted turtle	*Chrysemys picta*		XM_005286822.3
Gharial	*Gavialis gangeticus*		XM_019522525.1
Saltwater crocodile	*Crocodylus porosus*		XM_019554381.1
Western clawed frog	*Xenopus tropicalis*	Amphibia	NM_001011169.1
African clawed frog	*Xenopus laevis*		NM_001094219.1
Coelacanth	*Latimeria chalumnae*	Sarcopterygii	XM_014485595.1
Sailfin molly	*Poecilia latipinna*	Actinopterygii	XM_015022732.1
Amazon molly	*Poecilia formosa*		XM_007575256.2
Moon fish	*Xiphophorus maculatus*		XM_005815496.3
Beira killifish	*Nothobranchius kuhntae*		HAEE01001557.1
Sheepshead minnow	*Cyprinodon variegatus*		XM_015398436.1
Bicolor damselfish	*Stegastes partitus*		XM_008279449.1
Nile tilapia	*Oreochromis niloticus*		XM_003456202.5
Atlantic molly	*Poecilia mexicana*		XM_014990013.1
Cichlid fish	*Pundamilia nyererei*		XM_005751575.1
Great blue-spotted mudskipper	*Boleophthalmus pectinirostris*		XM_020933260.1
Ballan wrasse	*Labrus bergylta*		XM_020635481.1
Asian seabass	*Lates calcarifer*		XM_018696620.1

Asian swamp eel	*Monopterus albus*		XM_020606606.1
Japanese pufferfish	*Takifugu rubripes*		XM_011613800.1
Zebrafish	*Danio rerio*		BC162666.1
Catfish	*Ictalurus punctatus*		NM_001201086.2
Mexican tetra	*Astyanax mexicanus*		XM_007261051.3
Redbelly piranha	*Pygocentrus nattereri*		XM_017692226.1
American pike	*Esox lucius*		XM_010880216.3
Coast rainbow trout	*Oncorhynchus mykiss*		XM_021597675.1
Arowana	*Scleropages formosus*		XM_018732277.1
Spotted gar	*Lepisosteus oculatus*		XM_015358639.1
Zebra mbuna	*Maylandia zebra*		XM_004571956.2
Elephant fish	*Callorhinchus milii*	Chondrichthyes	XM_007896172.1
Whale shark	*Rhincodon typus*		XM_020532577.1

Accession numbers for vertebrate *Insig-1* genes

Common name	Species name	Class	Accession No.
Human	*Homo sapiens*	Mammalia	NM_001346591.1
Mississippi alligator	*Alligator mississippiensis*	Reptilia	XM_006275067.3
Zebrafish	*Danio rerio*	Actinopterygii	NM_199869.1
Elephant fish	*Callorhinchus milii*	Chondrichthyes	XM_007884440.1
Western clawed frog	*Xenopus tropicalis*	Amphibia	XM_012964967.2
Chicken	*Gallus gallus*	Aves	NM_001030966.1

Genome assemblies used to identify *Insig-2* genes

Common name	Species name	Class	Assembly ID	Scaffold	Start	End
Minke whale	*Balaenoptera acutorostrata*	Mammalia	GCA_000493695.1	NW_006725565.1	4972494	4960890

Antarctic minke whale	*Balaenoptera bonaerensis*		GCA_000978805.1	DF407697.1	1710	13242
Beluga whale	*Delphinapterus leucas*		GCA_002288925.2	NW_019160898.1	11223653	11212392
Harbour porpoise	*Phocoena phocoena*		GCA_003071005.1	PKGA01141833.1	2065978	2077444
Sperm whale	*Physeter catodon*		GCA_900411695.1	NW_019873604.1	5441934	5453423
Indo-Pacific bottlenose dolphin	*Tursiops aduncus*		GCA_003227395.1	NCQN01000653.1	398673	387374
slender-necked sea snake	*Hydrophis melanocephalus*	Reptilia	GCA_004320005.1	BHFS01009993.1	5048	13767

Genome and transcriptome assemblies used to identify *Insig-1/Insig-2* gene.

**Common name**	**Species name**	**Phylum**	**Assembly ID**	**Scaffold**	**Start**	**End**
Purple sea urchin	Strongylocentrotus purpuratus	Echinodermata	GCA_000002235.3	NW_011992292.1	5206	9925
				NW_011968172.1	188936	185481
Pacific oyster	crassostrea gigas	Mollusca	GCA_000297895.1	NW_011935183.1	372958	380017
Giant owl limpet	Lottia gigantea	Mollusca	GCA_000327385.1	NW_008710566.1	780987	785179
California two-spot octopus	*Octopus bimaculoides*	Mollusca	GCA_001194135.1	NW_014655479.1	83350	77766
Sea walnut	*Mnemiopsis leidyi*	Ctenophora	GCA_000226015.1	JH153334.1	192047	187117
Starlet sea anemone	*Nematostella vectensis*	Cnidaria	GCA_000209225.1	NW_001834356.1	898854	901029
Lamp shell	*Lingula anatina*	Brachiopoda	GCA_001039355.2	NW_019775336.1	343857	341715
Blood fluke	*Schistosoma mansoni*	Platyhelminthes	GCA_000237925.2	NC_031498.1	26028045	26027260
Sponge	*Amphimedon queenslandica*	Porifera	GCA_000090795.1	NW_003546250.1	169942	168648
Sea lamprey	*Petromyzon marinus*	Cyclostomata	GCA_002833325.1	PIZI01000005.1	7494194	7478459
				PIZI01000047.1	202581	206411
Florida lancelet	*Branchiostoma floridae*	Cephalochordata	GCA_000003815.1	NW_003101522.1	917404	915587
Sea squirt (**Not found**)	*Ciona intestinalis*	Tunicata	GCA_000224145.2			

Supplementary Table 2. Likelihood ratio tests of selective pressures on vertebrate *Insig-2* gene.

Model	ω value			ln *L*	Models compared	2Δ (ln *L*)	*P* value
	ω_0	ω_1	ω_2/ω_f				
**A: Free-ratio**	Variable ω by branch			-11551.498			
**B: One-ratio**	0.053			-11667.120	A vs. B	231.244	**0.008**
**C: Two-ratio**	0.058	0.046		-11665.385	B vs. C	3.470	0.062
**D: M2a_rel**	0.016 (83.4%)	1 (1.1%)	0.230 (15.5%)	-11421.236			
**E: CmC**	0.017 (83.7%)	1 (1.0%)	0.283 (15.3%)	-11415.937	D vs. E	10.598	**0.001**
			Terrestrial: 0.169				

Note- ω_f is the divergent site class that has a separate ω value.
