# Supplementary material for: Competitive oxidation and ubiquitylation on the evolutionarily conserved cysteine confer tissue-specific stabilization of Insig-2
Source: Nat Commun. 2020 Jan 17;11:379. doi: 10.1038/s41467-019-14231-w (PMC6969111; doi:10.1038/s41467-019-14231-w)
Supplement: Supplementary file 2 — Description of Additional Supplementary Files [file 41467_2019_14231_MOESM2_ESM.pdf]

Description of Additional Supplementary Files

File Name: Supplementary Data 1
Description: This file contains the Insig sequences of different species.
