# Supplementary material for: Competitive oxidation and ubiquitylation on the evolutionarily conserved cysteine confer tissue-specific stabilization of Insig-2
Source: Nat Commun. 2020 Jan 17;11:379. doi: 10.1038/s41467-019-14231-w (PMC6969111; doi:10.1038/s41467-019-14231-w)
Supplement: Supplementary file 4 — Reporting Summary [file 41467_2019_14231_MOESM4_ESM.pdf]

nature research

Corresponding author(s): Bao-Liang Song

Last updated by author(s): Dec 12, 2019

# Reporting Summary

Nature Research wishes to improve the reproducibility of the work that we publish. This form provides structure for consistency and transparency in reporting. For further information on Nature Research policies, see Authors & Referees and the Editorial Policy Checklist.

## Statistics

For all statistical analyses, confirm that the following items are present in the figure legend, table legend, main text, or Methods section.

| n/a | Confirmed | |
|---|---|---|
| ☐ | ☒ | The exact sample size (*n*) for each experimental group/condition, given as a discrete number and unit of measurement |
| ☐ | ☒ | A statement on whether measurements were taken from distinct samples or whether the same sample was measured repeatedly |
| ☐ | ☒ | The statistical test(s) used AND whether they are one- or two-sided<br>*Only common tests should be described solely by name; describe more complex techniques in the Methods section.* |
| ☒ | ☐ | A description of all covariates tested |
| ☒ | ☐ | A description of any assumptions or corrections, such as tests of normality and adjustment for multiple comparisons |
| ☐ | ☒ | A full description of the statistical parameters including central tendency (e.g. means) or other basic estimates (e.g. regression coefficient) AND variation (e.g. standard deviation) or associated estimates of uncertainty (e.g. confidence intervals) |
| ☒ | ☐ | For null hypothesis testing, the test statistic (e.g. *F*, *t*, *r*) with confidence intervals, effect sizes, degrees of freedom and *P* value noted<br>*Give P values as exact values whenever suitable.* |
| ☐ | ☒ | For Bayesian analysis, information on the choice of priors and Markov chain Monte Carlo settings |
| ☒ | ☐ | For hierarchical and complex designs, identification of the appropriate level for tests and full reporting of outcomes |
| ☒ | ☐ | Estimates of effect sizes (e.g. Cohen's *d*, Pearson's *r*), indicating how they were calculated |

*Our web collection on statistics for biologists contains articles on many of the points above.*

## Software and code

Policy information about availability of computer code

| | |
|---|---|
| Data collection | TBLASTN, CODEML, OLYMPUS BX53F, BECKMAN cytoflex, CytExpert 1.2, CFX Connect Real-Time System, Tanon-5200 |
| Data analysis | GraphPad Prism 6, ImageJ 1.45s, Microsoft Office Excel, PAML 4.7, MUSCLE, ModelTest, MrBayes v3.1.2, MEGA6 |

For manuscripts utilizing custom algorithms or software that are central to the research but not yet described in published literature, software must be made available to editors/reviewers. We strongly encourage code deposition in a community repository (e.g. GitHub). See the Nature Research guidelines for submitting code & software for further information.

## Data

Policy information about availability of data

All manuscripts must include a data availability statement. This statement should provide the following information, where applicable:

- Accession codes, unique identifiers, or web links for publicly available datasets
- A list of figures that have associated raw data
- A description of any restrictions on data availability

*Provide your data availability statement here.*

# Field-specific reporting

Please select the one below that is the best fit for your research. If you are not sure, read the appropriate sections before making your selection.

☒ Life sciences ☐ Behavioural & social sciences ☐ Ecological, evolutionary & environmental sciences

For a reference copy of the document with all sections, see nature.com/documents/nr-reporting-summary-flat.pdf

# Life sciences study design

All studies must disclose on these points even when the disclosure is negative.

| | |
|---|---|
| Sample size | No statistical methods were used to predetermine sample sizes. All sample numbers were stated in figure legends. |
| Data exclusions | No data was excluded from the analyses. |
| Replication | All attempts of replication were successful. We stated the number of replicates for each experiment in the figure legend. |
| Randomization | All cells and mice were allocated randomly. |
| Blinding | No blinding was performed in this study. |

# Reporting for specific materials, systems and methods

We require information from authors about some types of materials, experimental systems and methods used in many studies. Here, indicate whether each material, system or method listed is relevant to your study. If you are not sure if a list item applies to your research, read the appropriate section before selecting a response.

## Materials & experimental systems

| n/a | Involved in the study | |
|---|---|---|
| ☐ | ☒ | Antibodies |
| ☐ | ☒ | Eukaryotic cell lines |
| ☒ | ☐ | Palaeontology |
| ☐ | ☒ | Animals and other organisms |
| ☒ | ☐ | Human research participants |
| ☒ | ☐ | Clinical data |

## Methods

| n/a | Involved in the study | |
|---|---|---|
| ☒ | ☐ | ChIP-seq |
| ☐ | ☒ | Flow cytometry |
| ☒ | ☐ | MRI-based neuroimaging |

## Antibodies

| | |
|---|---|
| Antibodies used | All antibodies used in this study were stated in methods and figure legend.<br>mouse monoclonal anti-ubiquitin antibody P4D1 (SC-8017, Santa Cruz, 1:500);<br>mouse monoclonal anti-β-actin (A1978, Sigma, 1:10000);<br>rabbit polyclonal anti-Myc (06-549, Millipore, 1:1000);<br>rabbit polyclonal anti-GAPDH (10494-1-AP, Proteintech, 1:10000);<br>rabbit polyclonal anti-Insig-2, rabbit polyclonal anti-gp78 and mouse monoclonal anti-Myc (9E10) were prepared in our laboratory. |
| Validation | All the antibodies used in this paper were validated by immunoblotting and stated within the paper. |

## Eukaryotic cell lines

Policy information about cell lines

| | |
|---|---|
| Cell line source(s) | All cells used in this study were stated in the method of this paper. CHO-7,1601 and C2C12 cells were obtained from ATCC. Muscle stem cells were obtained from mice muscle |
| Authentication | No further authentication of the cell lines was performed before use. |
| Mycoplasma contamination | No test for mycoplasma contamination was performed. |
| Commonly misidentified lines (See ICLAC register) | None of these cell lines were utilized. |

## Animals and other organisms

Policy information about studies involving animals; ARRIVE guidelines recommended for reporting animal research

| | |
|---|---|
| Laboratory animals | All mice used in this study were stated in method and figure legend. Male, 8 week old C57BL/6J mice gp78-/- mice and Insig-2-/- mice were used. |
| Wild animals | The study did not involve wild animals. |

| | |
|---|---|
| Field-collected samples | The study did not involve field-collected samples. |
| Ethics oversight | All animals were maintained and used in accordance with the guidelines of the Institutional Animal Care and Use Committee of the Wuhan university. |

Note that full information on the approval of the study protocol must also be provided in the manuscript.

# Flow Cytometry

## Plots

Confirm that:

- [x] The axis labels state the marker and fluorochrome used (e.g. CD4-FITC).
- [ ] The axis scales are clearly visible. Include numbers along axes only for bottom left plot of group (a 'group' is an analysis of identical markers).
- [ ] All plots are contour plots with outliers or pseudocolor plots.
- [ ] A numerical value for number of cells or percentage (with statistics) is provided.

## Methodology

| | |
|---|---|
| Sample preparation | Intracellular ROS level was determined using oxidation-sensitive DCFH-DA fluorescent dyes. Cells were washed twice with PBS and labeled on the culture plates with DCFH-DA for 30 min at 37 °C in serum-free medium. At the end of incubation, culture plates were trypsinized, resuspended in PBS and analyzed using a FACScan flow cytometer. |
| Instrument | BECKMAN cytoflex |
| Software | CytExpert 1.2 |
| Cell population abundance | The mean fluorescence intensity of 10,000 cells was measured in each sample and corrected for autofluorescence from unlabeled cells. |
| Gating strategy | *Describe the gating strategy used for all relevant experiments, specifying the preliminary FSC/SSC gates of the starting cell population, indicating where boundaries between "positive" and "negative" staining cell populations are defined.* |

- [ ] Tick this box to confirm that a figure exemplifying the gating strategy is provided in the Supplementary Information.
